# Impact of pH modification of the empirically used tobramycin ophthalmic solution on MIC90 concentration in tears and aqueous humor of donkeys (*Equus asinus*)

**DOI:** 10.1186/s12917-024-04072-1

**Published:** 2024-05-23

**Authors:** Ahmed Ibrahim, Mohamed A. A. Abd-Elrasoul, Mahmoud S. Sabra

**Affiliations:** 1 Veterinary Teaching Hospital, Faculty of Veterinary Medicine, Assiut University, Assiut, 71526 Egypt; 2 Central Laboratory, Faculty of Veterinary Medicine, Assiut University, Assiut, 71526 Egypt; 3 Department of Pharmacology, Faculty of Veterinary Medicine, Assiut University, Assiut, 71526 Egypt

**Keywords:** Keratitis, Topical antibiotics, Tobramycin, Drug bioavailability, Equines, Eyes

## Abstract

**Background:**

Commercial tobramycin ophthalmic solution is frequently used empirically to treat ocular disorders in equines, despite being primarily formulated for use in humans. It has been noted that tobramycin MIC90 concentration (minimal inhibitory concentration to 90% of microbial growth) rapidly declined following topical administration. It is hypothesized that adjustment of the pH of the empirically used tobramycin ophthalmic solution -prepared for human use- with the pH of the tears of donkeys, could increase the bioavailability of the drug and subsequently improve its penetration to the aqueous humor. Therefore, this study aimed to evaluate the impact of pH adjustment of the empirically used tobramycin ophthalmic solution on MIC90 concentration in tears and aqueous humor of donkeys (*Equus asinus*). The study was conducted on six (*n* = 6) clinically healthy donkeys. In each donkey, one eye was randomly selected to receive 210 µg tobramycin of the commercial tobramycin (CT) and used as a positive control (C group, *n* = 6). The other eye (treated eye) received 210 µg of the modified tobramycin ophthalmic solution (MT) (T group, *n* = 6). Tears and aqueous humor samples were collected 5-, 10-, 15-, 30- min, and 1-, 2-, 4-, and 6 h post-instillation.

**Results:**

Modifying the pH of the empirically used commercial tobramycin ophthalmic solution in donkeys at a pH of 8.26 enhanced the drug’s bioavailability. The MIC90 of the most hazardous bacteria isolated from equines’ eyes such as *Pseudomonas aeruginosa* (MIC90 = 128 µg/ml) and *Staphylococcus aureus* (MIC90 = 256 µg/ml) was covered early (5 min post-instillation) and over a longer period in donkey tears (239–342 min) and aqueous humor (238–330 min) with the modified tobramycin solution.

**Conclusions:**

Adjustment of the pH of the commercial tobramycin ophthalmic solution, empirically used by veterinarians to treat donkeys’ ophthalmic infections at a pH of 8.26, isotonic with the donkeys’ tears pH, resulting in higher concentrations of tobramycin in tears and aqueous humor for a longer time.

**Supplementary Information:**

The online version contains supplementary material available at 10.1186/s12917-024-04072-1.

## Background

Sight is a vital means by which the animal explores and interacts with its environmental surroundings; therefore, it is crucial to provide good eye care for it [1]. Equines are characterized by the prominence of the eyeball. This increases the risk of ocular trauma and infections [1, 2]. Like horses, bacterial keratitis, conjunctivitis, and uveitis have been recorded in donkeys [1–5]. Staphylococcus, Streptococcus, and Pseudomonas sp. were the most common bacterial pathogens isolated in cases of equine corneal ulcers [6, 7]. Inadequate treatment of such ocular disorders could worsen vision and even lead to blindness [2, 3]. In most cases, proper treatment of these ocular affections requires efficient topical antibiotics [8].

Most described topical ophthalmic regimens used for treating equine individuals with ocular diseases are primarily formulated for use in humans [9, 10]. Therefore, topical tobramycin is frequently used empirically for the treatment of ocular disorders in equines [11]. Tobramycin is a broad-spectrum aminoglycoside antibiotic [12]. It has bactericidal activity mainly on gram-negative bacteria. Tobramycin is actively transported through the bacterial cell membrane. Then it inactivates the initiation complex of the translation process by inhibiting protein production through irreversible binding to the 30 S and 50 S ribosomal subunits [13].

The pharmacokinetics of the ophthalmic tobramycin drops in horses have been addressed in a previous limited literature. Equine eye bacteria, including *Pseudomonas aeruginosa* (MIC90 = 128 µg/ml) and *Staphylococcus* aureus (MIC90 = 256 µg/ml), are among the most dangerous pathogens. It has been noted that tobramycin MIC90 concentration (minimal inhibitory concentration to 90% of microbial growth) in tears and in vitro declines rapidly following topical administration [14–16]. A topical single dose of tobramycin gives rise to a higher therapeutic concentration in the tears of horses for 1 h (h) after administration with a concentration kept above the MIC90 for mostly isolated bacteria for 68.5 min (min) [11]. However, these levels were maintained in equine tears 6 times longer than in rabbit tears [15].

The ophthalmic drug bioavailability could be improved by increasing contact time in the cul-de-sac and/or increasing the ocular membrane permeability [17]. Therefore, the ideal ophthalmic medications for topical ocular delivery should be isotonic with tears for comfort and drug stability [18]. The difference in the pH often limits the quickness and the quantity of drug absorbed [19]. Because of the differences in the pH of tear fluids between humans and equines [9, 20], it has appeared that the optimal ocular pH for treated animals will not be the same as that for humans [9].

The authors’ hypothesis was that adjustment of the pH of the empirically used tobramycin ophthalmic solution -prepared for human use- with the pH of the tears of donkeys, could increase the bioavailability of the drug and subsequently improve its penetration to the aqueous humor. Therefore, the study aimed to evaluate the impact of pH adjustment of the empirically used tobramycin ophthalmic solution on MIC90 concentration in tears and aqueous humor of donkeys (*Equus asinus*).

## Methods

### Ethical approval

All the procedures in this study have been approved by the Research Ethics Committee (REC) of the Faculty of Veterinary Medicine, Assiut University, Assiut, Egypt, in compliance with Egyptian bylaws and the OIE animal welfare standards for the care and use of animals in research and education, under the No. (06/2023/0041). The procedures were carried out in compliance with relevant guidelines and regulations.

### Drugs

#### Commercial tobramycin ophthalmic solution (CT)

Tobramycin 0.3% (Tobrex®, sterile ophthalmic solution, 5 ml, each ml contains tobramycin 3 mg, Alcon-Couvreur NV, Puurs, 2870, Belgium).

#### Measurement of pH of the CT

The commercial ophthalmic tobramycin solution pH was measured using a digital pH meter with a range of 0–14 (Qis Proline pH B210, India). The glass microelectrode of the pH meter was inserted in a test tube containing the tobramycin solution in a manner that covered its tip.

#### Measurement of pH of the donkeys’ tears

pH of a collected tear sample on day 0 (before instillation) was measured using a digital pH meter as described before.

#### Modification of pH of the CT

The modification of the pH of the CT was done under strict sterilized conditions (Microzone BioKlone, Model BK-2-6, US). Phosphate buffer saline pH 9 was titrated drop by drop into the commercial tobramycin solution until reaching a pH matched with the pH of the donkeys’ tears.

### Animals

The study was conducted on six (*n* = 6) clinically healthy donkeys (*Equus asinus*) of both sexes (3 males and 3 non-pregnant, non-lactating females), aged 3 − 5 years, and weighing 150–200 kg. Donkeys were obtained from the Animal Research Unit - Veterinary Teaching Hospital - Faculty of Veterinary Medicine – Assiut - Egypt. Donkeys were housed indoors to avoid bright sunlight, dust, and wind, with feed and water *ad libitum*. Donkeys were included in the study if they had no ocular disorders based on complete ophthalmic examination including, Schirmer tear test (STT), slit lamp biomicroscopy, indirect ophthalmoscopy, tonometry, and fluorescein test and received no systemic or topical antibiotics for the last three months. Animals got an acclimatization period of one week before the experiment.

### Experimental design

In each donkey, one eye was randomly selected to receive two drops (70 µL = 210 µg tobramycin) of the CT and used as a positive control (C group, *n* = 6 eyes). The other eye (treated eye) received 210 µg (140 µL = 4 drops) of the modified tobramycin ophthalmic solution (MT) (T group, *n* = 6). This selection was kept throughout the study.

Each eye was used for a single collection of tears and aqueous humor per time point each day followed by a wash period of 24 h. Tears and aqueous humor samples were collected 5-, 10-, 15-, 30- min, and 1-, 2-, 4-, and 6 h post-instillation at a fixed time of the day (8:00) AM.

### Tear collection

**Fig. 1:**
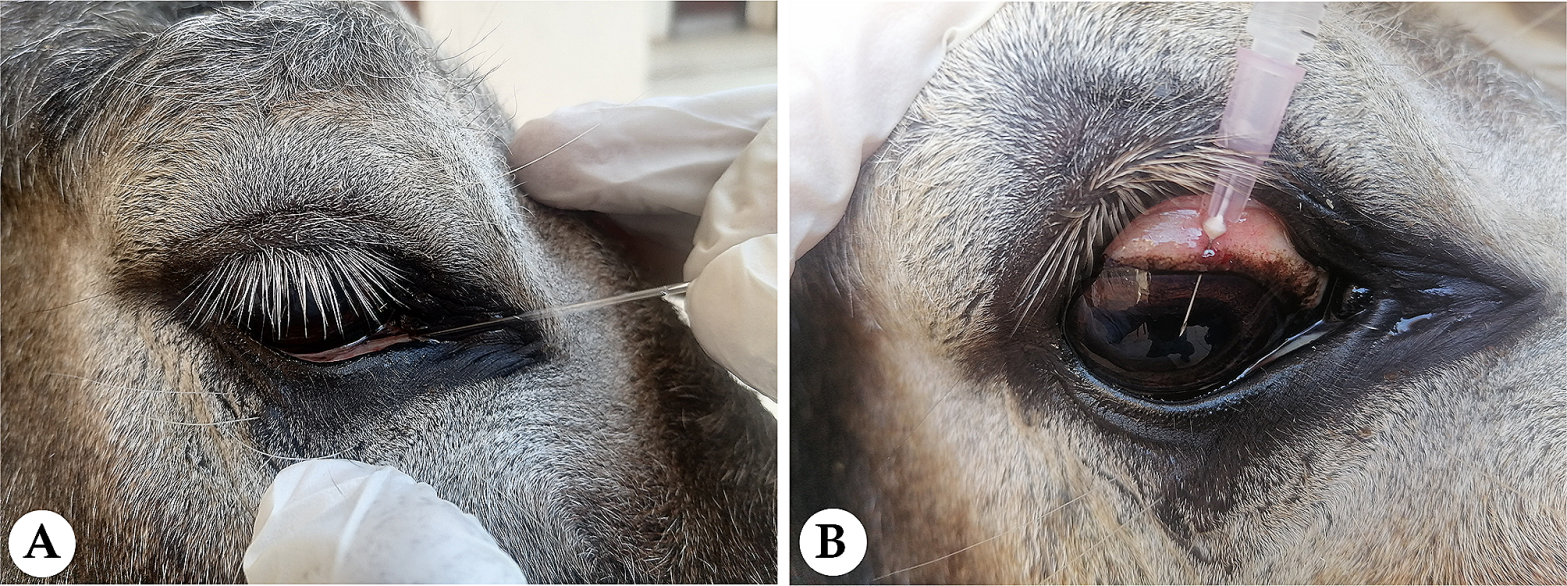
(**A**) The capillary tube was inserted into the ventral conjunctival fornix at the medial canthus of the eye to collect tear samples in donkeys. (**B**) A sterile syringe with a 30-G needle was inserted into the anterior chamber at the limbus to collect an aqueous humor sample from the donkeys

Glass capillary tubes were used for the collection of tear samples. Donkeys received intravenous (IV) 2% xylazine HCl (1.1 mg/kg, Xyla-Ject, ADWIA Co., SAE, Egypt) for tranquilization. Under the physical restrain, the lower eyelid was gently pulled downward, and the capillary tube was inserted into the ventral cul-de-sac at the medial canthus of the eye. The capillary tube was held horizontally and somewhat laterally without contact with the cornea or conjunctiva (Fig. 1A). Tears are sucked into the capillary tube by the adhesive forces acting upon the surface of the inside of the hollow cylinder. The tear fluid (100 µL) was then pipetted out into the Eppendorf tube via blowing the air into the capillary tube by a 200 µL Eppendorf pipette and stored at -80 ℃ until analysis. Tear samples were analyzed within 30 days of collection.

### Aqueous humor collection

The aqueous humor sample (100 µL) was collected from the eye via anterior chamber paracentesis. Donkeys received intravenous (IV) 2% xylazine HCl (1.1 mg/kg, Xyla-Ject, ADWIA Co., SAE, Egypt) and 5% Ketamine HCl (2.2 mg/kg, Ketamine, Sigma-tec Pharmaceutical Industries, SAE, Egypt). The ocular surface was then desensitized by two drops of 0.4% benoxinate hydrochloride ophthalmic solution (Benox, sterile ophthalmic solution, 10 ml, Egyptian International Pharmaceutical Industries Company, EPICO, Egypt). A sterile 1-mL insulin syringe with a 30-G needle was inserted into the anterior chamber at the limbus parallel to the iris with the bevel facing upwards (Fig. 1B). The samples were stored in Eppendorf tubes at -80 ℃ until analysis. Aqueous humor samples were analyzed within 30 days of collection.

### Drug assay

**Fig. 2:**
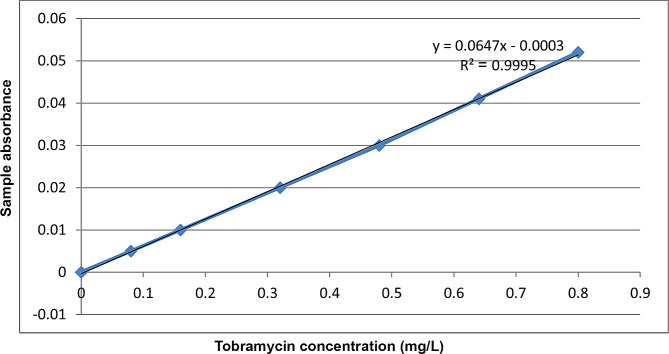
Tobramycin standard curve at 530 nm wavelength. Tobramycin concentration (mg/ml) is represented on the X-axis. The sample absorbance is shown on the Y-axis

The tears and aqueous humor samples were analyzed for tobramycin concentration. Tobramycin stock standard solution was made by dissolving 0.2 gram of standard tobramycin in 15 ml of distal water and then completing the volume to 25 ml with distal water in a 25 ml volumetric flask where the final concentration was (each 1 ml contains 8 mg tobramycin). Different quantities of the produced solution were taken (10 µl, 20 µl, 40 µl, 60 µl, 80 µl and 100 µl). The calibration curve was then created by serially diluting the stock standard solution (1 ml = 8 mg tobramycin). According to [32], tobramycin concentrations in tear and aqueous humor samples were evaluated by spectrophotometry using 1 ml ascorbic acid 0.1% in dimethyl sulfoxide (DMSO) added to each volume and boiling in a water bath for 30 min. At 530 nm, we computed the tobramycin concentration using the standard curve equation (Fig. 2).

### Statistical analysis

Statistical significance was assessed by one-way ANOVA for repeated measures, or two-way ANOVA, as appropriate. The Dunnett test and Bonferroni’s multiple comparisons test were used for data point comparisons in each group. *P* ≤ 0.05 was considered statistically significant. Data are presented as means ± SE. Graph Pad prism® software (version 8) was used to carry out these statistical tests.

## Results

Topical administration of the modified tobramycin ophthalmic solution (MT) was well tolerated by all donkeys, with no apparent reflex tearing. The tears pH of the donkeys (8.3) was different from the pH of the commercial topical tobramycin (7.24). The pH of the MT was adjusted at 8.26 with double the volume of the commercial one.

**Fig. 3:**
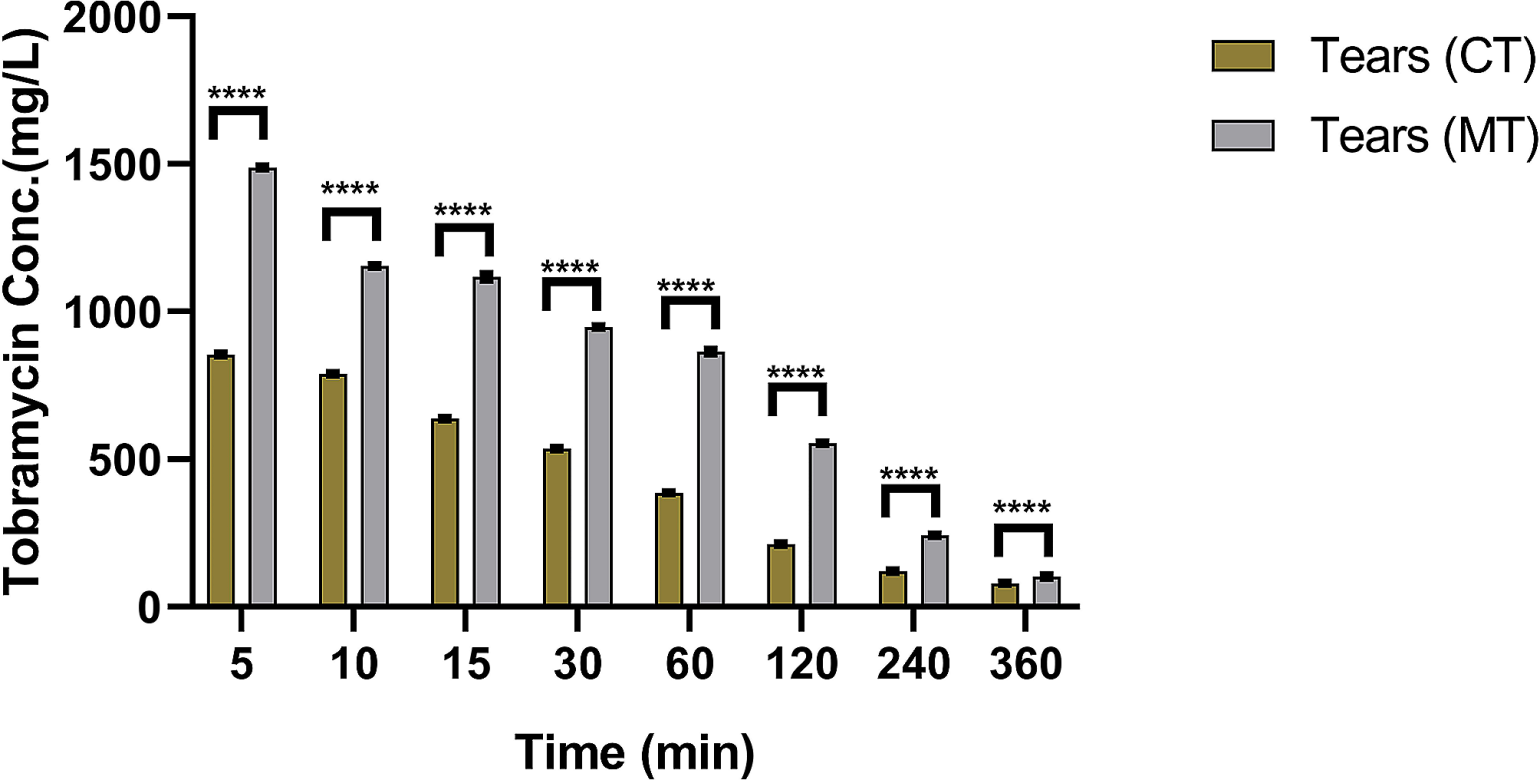
Tobramycin concentration in donkey tears at various periods for both commercial (CT) and modified (MT) tobramycin ophthalmic solution

Tears recorded highly significant concentrations of MT more than CT throughout the study at different time intervals (*P* < 0.05). There was a significant decrease in the concentrations of the MT and the CT following the first five minutes post-installation in the treated and control eyes’ tears, respectively (*P* < 0.05). Tobramycin MIC for *Pseudomonas aeruginosa* (MIC90 = 128 µg/ml) and *Staphylococcus aureus* (MIC90 = 256 µg/ml) was maintained longer in the treated group with MT (342 min) than in the CT (239 min) (Fig. 3).

**Fig. 4:**
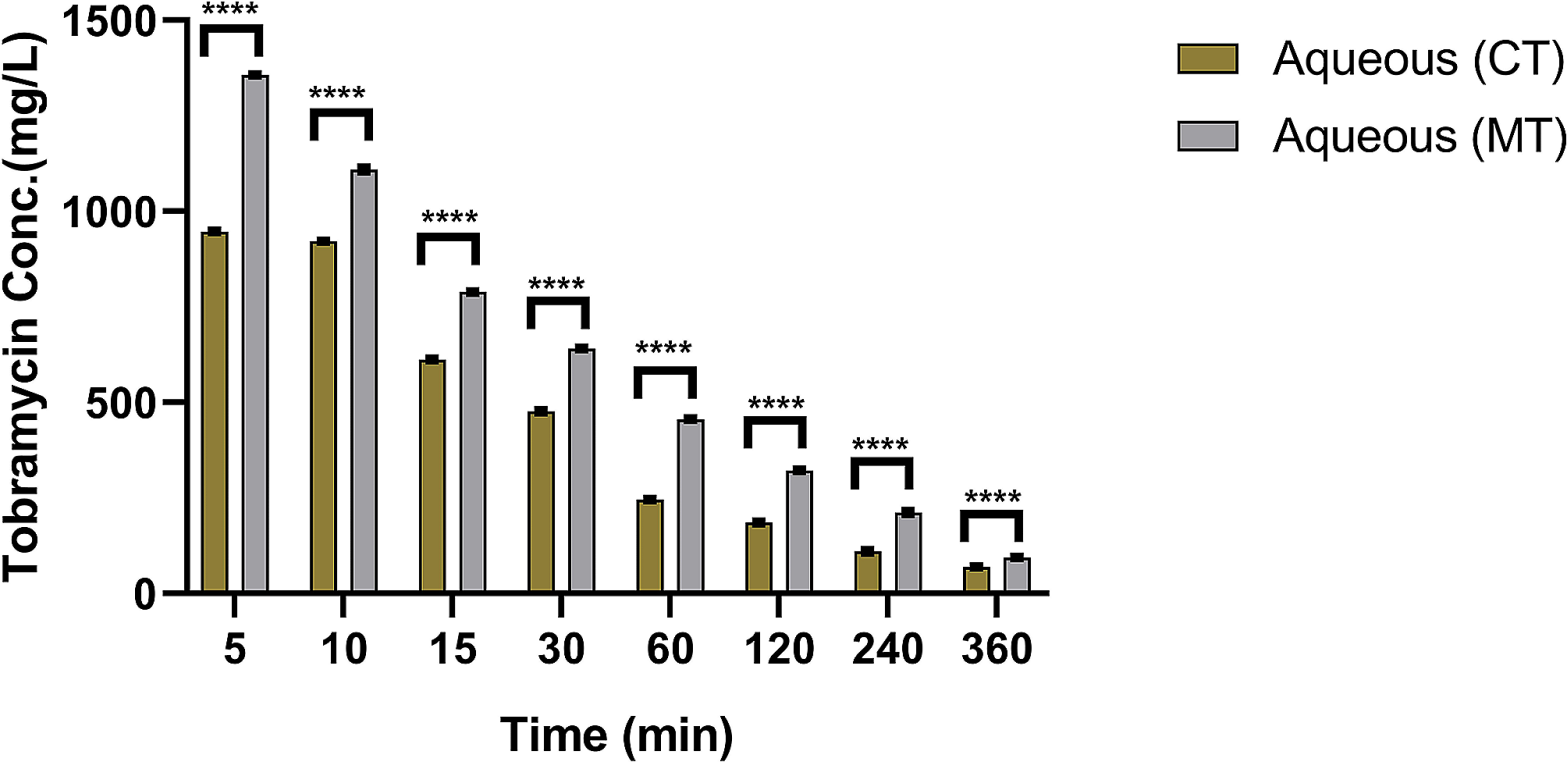
Tobramycin concentration in donkey aqueous humor at various periods for both commercial (CT) and modified (MT) tobramycin ophthalmic solution

Aqueous humor (AH) recorded highly significant (*P* < 0.05) concentrations of MT more than CT throughout the study at different time intervals parallel to the concentrations of the MT and CT in the tears of the treated and control eyes, respectively. Both MT and CT concentrations in the aqueous humor decreased significantly (*P* < 0.05) in treated and control eyes, respectively. Tobramycin MIC for *Pseudomonas aeruginosa* (MIC90 = 128 µg/ml) and *Staphylococcus aureus* (MIC90 = 256 µg/ml) sustained in the aqueous humor longer in the eyes received MT (330 min post instillation) compared with 238 min post instillation in the eyes received the CT (Fig. 4).

## Discussion

The findings of this study investigated whether changing the pH of an empirically used commercial tobramycin ophthalmic solution in donkeys to 8.26 increased the drug’s bioavailability. The MIC90 of the most dangerous bacteria isolated from equines’ eyes (128 g/ml) was covered in donkey tears (239 min for CT- 342 min for MT) and aqueous humor (238 min for CT-330 min for MT), with modified tobramycin solution superiority and longer duration.

Although donkeys’ eyeballs are usually slightly more sunken than horses, their thicker periocular hair and bigger conjunctival sac -compared with horses- provide an ideal environment for foreign bodies. Moreover, the corneal surface of donkeys is slightly larger and more convex than horses. In addition to the aforementioned factors, delayed behavioral responses (blepharospasm, epiphora, photophobia) in donkeys predispose donkeys to corneal ulceration [2].

Different techniques for tear sampling have been described, including Schirmer strips, capillary tubes, and surgical sponges [21]. Here, tear samples were collected using capillary tubes. Insertion of the capillary tubes into the ventral fornix at the medial canthus of the eye, where excess tears are normally collected to pass into the nasolacrimal drainage system, facilitated tear sampling [1]. Moreover, the Schirmer strips or surgical sponges require a further centrifugation process to extract the tear fluid from them with a small final volume of tears (1–2 µL). Whereas tear fluid is pipetted out of the capillary tubes directly into Eppendorf tubes after collection with a sufficient tear volume (100 µL). Therefore, capillary tubes seem to be simpler, quicker, and more practical than other techniques [21].

Donkeys received adequate tranquilization and anesthesia during tear and aqueous humor sampling to minimize the risk of ocular trauma that could result from a sudden movement of the animal’s head. It was found that tear production was not affected by xylazine HCl administration [22]. According to another study, the combination of xylazine HCL and ketamine HCL will not have a significant enough impact to harm the eye’s surface or tear production [23]. Various fields of ophthalmology, otology, rhinology, and laryngology make use of benoxinate hydrochloride. In particular, it works well for brief ophthalmologic operations including tonometry, fitting contact lenses, local analgesia of the wounded eye, and small eye surgeries [24].

Donkeys were housed indoors to limit exposure to sunlight, wind, and dust, which could affect medicine delivery to the eyes by increasing tear production and drug loss [25].

Tobramycin is a cationic hydrophilic member of aminoglycosides. It is used to treat many bacterial infections, notably Gram-negative organisms such as Pseudomonas strains. Tobramycin inhibits the development of the 70 S complex by targeting aminoglycoside receptors on 30 S and 50 S bacterial ribosomes. As a result, mRNA cannot be translated into proteins that confirm apoptosis and necrosis [26]. Topical instillation of 0.3% eyedrops in rabbit eyes on a loading dosage schedule resulted in fast drug penetration into the aqueous humor, with a peak of 3.24 mg/L at 2-hour intervals and beneficial values lasting up to 6 h [27].

The normal physiological pH of tear fluid in humans is 7.4. The pH comfort zone for topically applied ocular medicine ranges from 6.5 to 7.8. Therefore, drug instillation either in acidic or alkaline forms results in increasing tear secretion and loss of drug [9, 28]. The pH of horse tears was reported to be 7.84 ± 0.30 [9, 29]. In the current study, donkeys tolerated the pH-adjusted topical tobramycin without obvious reflex lacrimation. This is consistent with the finding of Fiscella [30] who stated that the closer the pH of the ophthalmic preparation is to tear pH, the less irritating the solution is to the eye. Our results aligned with those of Beckwith-Cohen et al. [9], who observed that horses have a higher alkaline comfort zone than humans.

The pH of commercial tobramycin was 7.24 in the current investigation and rose to 8.26 following modification. Also, a considerable increase in drug bioavailability in both aqueous humor and tears was seen 5 min after drug instillation and declined progressively over time, with the modified tobramycin solution covering the MIC90 of the most harmful isolated bacteria over a longer period. The MIC90 for tobramycin varied from 0.25 µg/ml to 256 µg/ml [31]. In the current study, both commercial and modified tobramycin concentrations were above the MIC90 of the most dangerous bacteria, such as *Pseudomonas aeruginosa* (MIC90 = 128 µg/ml) and *Staphylococcus aureus* (MIC90 = 256 µg/ml), with the modified tobramycin solution outperforming the commercial tobramycin solution.

## Conclusions

Modifying the pH of the empirically used commercial tobramycin ophthalmic solution in donkeys at a pH of 8.26 enhanced the drug’s bioavailability. The MIC90 of the most hazardous bacteria isolated from equines’ eyes (128 µg/ml) was covered early (5 min post-instillation) and for a longer period in donkey tears (239–342 min) and aqueous humor (238–330 min) with the modified tobramycin solution.

### Electronic supplementary material

Below is the link to the electronic supplementary material.

Supplementary Material 1

## Data Availability

All data generated or analyzed during this study are included in this published article
