# Supplementary material for: Impact of pH modification of the empirically used tobramycin ophthalmic solution on MIC90 concentration in tears and aqueous humor of donkeys (Equus asinus)
Source: BMC Vet Res. 2024 May 23;20:218. doi: 10.1186/s12917-024-04072-1 (PMC11112948; doi:10.1186/s12917-024-04072-1)
Supplement: Supplementary file 1 — Supplementary Material 1 [file 12917_2024_4072_MOESM1_ESM.docx]

| **MT** | **CT** | **Time (min)** |
| --- | --- | --- |
| 1488±2.31 ^b, A^ | 854.5±2.23 ^a, A^ | **5** |
| 1154±1.74 ^b, B^ | 789.5±1.74 ^a, B^ | **10** |
| 1117±8.531 ^b, C^ | 637.2±2.197 ^a, C^ | **15** |
| 948.2±1.662 ^b, D^ | 536.2±1.74 ^a, D^ | **30** |
| 864±4.662 ^b, E^ | 386.6±1.486 ^a, E^ | **60** |
| 553.5±1.727 ^b, F^ | 212.7±1.82 ^a, F^ | **120** |
| 242.5±1.83 ^b, G^ | 120.7±1.43 ^a, G^ | **240** |
| 102.2±3.13 ^b, H^ | 79.67±1.28 ^a, H^ | **360** |

**Table 1:** Tobramycin concentrations in tears of donkeys at different time intervals exposed to conventional (CT) and modified tobramycin medication (MT).

Different small superscript letters represent statistical differences (P < 0.05) on the same row at various time periods.

Different large superscript letters show statistically significant (P < 0.05) differences on the same column at various time periods.

**Table 2:** Tobramycin concentrations in aqueous humor of donkeys at different time intervals exposed to conventional (CT) and modified tobramycin medication (MT).

| **MT** | **CT** | **Time (min)** |
| --- | --- | --- |
| 1357±1.745^b, A^ | 947*.*3±2.29^a, A^ | **5** |
| 1109±4.828^b, B^ | 921.7±1.256^a, B^ | **10** |
| 789±2.066^b, C^ | 612.3±1.606^a, C^ | **15** |
| 641±1.653^b, D^ | 477±2.23^a, D^ | **30** |
| 455.8±1.701^b, E^ | 245.7±1.726^a, E^ | **60** |
| 322±1.98 ^b, F^ | 185.5±2.23^a, F^ | **120** |
| 212±3.47^b, G^ | 110.2±3.09^a, G^ | **240** |
| 94.5±1.56^b, H^ | 69.67±0.98^a, H^ | **360** |

Different small superscript letters represent statistical differences (P < 0.05) on the same row at various time periods.

Different large superscript letters show statistically significant (P < 0.05) differences in the same column at various time periods.
